# Host Transcriptional Signatures Predict Etiology in Community-Acquired Pneumonia: Potential Antibiotic Stewardship Tools

**DOI:** 10.1177/11772719221099130

**Published:** 2022-06-06

**Authors:** William W Siljan, Dhanasekaran Sivakumaran, Christian Ritz, Synne Jenum, Tom HM Ottenhoff, Elling Ulvestad, Jan C Holter, Lars Heggelund, Harleen MS Grewal

**Affiliations:** 1 Department of Pulmonary Medicine, Division of Medicine, Akershus University Hospital, Lørenskog, Norway; 2 Department of Clinical Science, Bergen Integrated Diagnostic Stewardship Cluster, Faculty of Medicine, University of Bergen, Bergen, Norway; 3 Department of Microbiology, Haukeland University Hospital, Bergen, Norway; 4 Department of Nutrition, Exercise and Sports, University of Copenhagen, Copenhagen, Denmark; 5 Department of Infectious Diseases, Oslo University Hospital, Oslo, Norway; 6 Department of Infectious Diseases, Leiden University Medical Center, Leiden, The Netherlands; 7 Department of Microbiology, Oslo University Hospital, Oslo, Norway; 8 Institute of Clinical Medicine, Faculty of Medicine, University of Oslo, Oslo, Norway; 9 Department of Internal Medicine, Vestre Viken Hospital Trust, Drammen, Norway

**Keywords:** Pneumonia, gene expression signatures, bacteria, viruses, antimicrobial stewardship, clinical decision-making

## Abstract

**Background:**

Current approaches for pathogen identification in community-acquired pneumonia (CAP) remain suboptimal, leaving most patients without a microbiological diagnosis. If better diagnostic tools were available for differentiating between viral and bacterial CAP, unnecessary antibacterial therapy could be avoided in viral CAP patients.

**Methods:**

In 156 adults hospitalized with CAP classified to have bacterial, viral, or mixed viral-bacterial infection based on microbiological testing or both microbiological testing and procalcitonin (PCT) levels, we aimed to identify discriminatory host transcriptional signatures in peripheral blood samples acquired at hospital admission, by applying Dual-color-Reverse-Transcriptase-Multiplex-Ligation-dependent-Probe-Amplification (dc-RT MLPA).

**Results:**

In patients classified by microbiological testing, a 9-transcript signature showed high accuracy for discriminating bacterial from viral CAP (AUC 0.91, 95% CI 0.85-0.96), while a 10-transcript signature similarly discriminated mixed viral-bacterial from viral CAP (AUC 0.91, 95% CI 0.86-0.96). In patients classified by both microbiological testing and PCT levels, a 13-transcript signature showed excellent accuracy for discriminating bacterial from viral CAP (AUC 1.00, 95% CI 1.00-1.00), while a 7-transcript signature similarly discriminated mixed viral-bacterial from viral CAP (AUC 0.93, 95% CI 0.87-0.98).

**Conclusion:**

Our findings support host transcriptional signatures in peripheral blood samples as a potential tool for guiding clinical decision-making and antibiotic stewardship in CAP.

## Introduction

Community-acquired pneumonia (CAP) is responsible for considerable morbidity and mortality across all ages and continents.^1^ The incidence of CAP and the rates of hospitalizations and admissions to intensive care units (ICUs), are expected to increase in the forthcoming years due to demographic trends, especially in Western societies.^2^ Previously, bacterial pathogens have been considered the principal cause of CAP and subsequently, empirical antibiotic therapy has been the backbone of CAP management. However, in recent years, advances in molecular diagnostic techniques (eg, nucleic acid amplification tests [NAATs]) have enhanced the ability to detect viruses in respiratory samples, suggesting a causative viral pathogen in up to one-third of adult CAP patients.^3^ With rapid and reliable discrimination between viral and bacterial CAP, antibiotic therapy could be avoided in many patients,^4^ counteracting one of the major challenges of our time; increasing antibiotic resistance associated with inappropriate overuse. In addition, at the individual level, antibiotics may cause adverse drug events and alter the human gut microbiome, paving the way for *Clostridium difficile* infections, increasing mortality, morbidity, length of hospital stay and costs of care.^5,6^ Appropriate sampling from the lower respiratory tract is ideal but challenging, limited by invasive techniques and the risk of contamination from both the nasal and oropharyngeal microbiota.^7^ In comparison, routinely available peripheral blood markers of inflammation (eg, C-reactive protein [CRP] and procalcitonin [PCT]) are not precise enough to independently discriminate CAP caused by (i) bacteria, (ii) viruses, or (iii) mixed viral-bacterial infections, although these are established biomarkers for differentiating bacterial from viral infections.^4,8^ A reliable diagnostic tool for this purpose would allow more tailored antimicrobial therapy and have a positive impact on antibiotic stewardship.^9^

Intriguingly, RNA profiling of human whole blood (WB) in various infections suggests that the lack of specificity obtained for single inflammatory markers can be compensated by the expression patterns constituted by multiple genes.^10^ Gene signatures providing high- to- excellent discriminatory accuracy between bacterial and viral respiratory infections have been identified in adults.^11-14^ Microarray and RNA sequencing techniques are useful for the unbiased discovery of novel biomarkers/biosignatures but are technically demanding, costly, and complicated by extensive data analysis. Dual-color-Reverse-Transcriptase-Multiplex-Ligation-dependent-Probe-Amplification (dc-RT MLPA) is a robust, low-cost multiplexed RT-PCR based technique with a dynamic range and sensitivity comparable to real-time quantitative polymerase chain reaction (PCR) and RNA sequencing, suited to determine host transcription signatures based on a more limited number of genes in larger populations.^15^ The selection of genes for the dc-RT MLPA platform covers several mediators of innate, adaptive and inflammatory immunity, including myeloid cell activation, Th1/Th2-responses, and type 1-interferon inducible genes relevant to respiratory infections.^16,17^

In a well-defined cohort of 156 adults hospitalized with CAP and classified to have (i) bacterial, (ii) viral, or (iii) mixed viral-bacterial infection established through an extensive microbiological work-up,^18^ we aimed to identify discriminatory transcriptional signatures in peripheral WB samples by the dc-RT MLPA, in order to identify patients where antibiotic treatment could safely be retained. We further hypothesized that the combined use of extensive microbiological testing and PCT could provide an even more robust classification of CAP etiology, associated with particularly distinct immune profiles, and thus enhance the discriminatory accuracy of host transcriptional signatures.

## Materials and Methods

### Study population and design

This study is an analysis of samples obtained from a prospective cohort study designed to establish the microbial etiology in hospitalized patients with CAP and identify risk factors for adverse outcome (NCT01563315).^18^ It was carried out in Drammen, Vestre Viken Hospital Trust, serving a catchment population of 160 000 in South-Eastern Norway. Adult patients (aged ⩾18 years) with suspected pneumonia who were admitted between January 1st 2008 and January 31st 2011 to the Medical Department were consecutively recruited and screened for inclusion within 48 hours. CAP was defined as (i) the presence of a new pulmonary infiltrate on chest radiograph, (ii) rectal temperature >38.0°C, and (iii) at least 1 of the following symptoms or signs: cough (productive or non-productive), dyspnea, respiratory chest pain, crackles, or reduced respiratory sounds. Exclusion criteria were: (i) chest radiograph showed non-infectious cause for pulmonary infiltrates such as pulmonary infarction, tumor or bronchiectasis and (ii) hospitalization within past 2 weeks. The inclusion process is summarized in Supplemental Text 1.

All patients provided written informed consent. The study was approved by the Regional Committee for Medical and Health Research Ethics in South-Eastern Norway (reference number: S-06266a).

### Data collection and microbiological sampling

Demographic, clinical and laboratory data were collected within 48 hours of admission. The microbial etiology of CAP was established by extensive microbiological testing (Supplemental Table 1). A complete sample collection constituted the collection of blood, sputum and nasopharyngeal samples for culture; nasopharyngeal and oropharyngeal samples analyzed for *Streptococcus pneumoniae*, *Mycoplasma pneumoniae*, *Chlamydophila pneumoniae*, *Bordetella pertussis*, and 12 types of respiratory viruses by use of PCR; serological testing for *Mycoplasma pneumoniae*, *Chlamydophila pneumoniae*, *Bordetella pertussis*, and influenza A and B viruses; and urine antigen assays for detection of *Streptococcus pneumoniae* and *Legionella pneumophila* antigens. Etiology was considered to be definite or probable, based on predefined criteria, as specified in detail previously.^18^

### Selection of transcriptional biomarkers

A total of 156 genes (including 4 housekeeping genes), distributed in 2 panels, were used in the dc-RT MLPA (Supplemental Table 2). The first 92-gene panel included genes involved in general inflammation, myeloid cell activation, and adaptive immunity, comprising of Th1/Th2-responses, regulatory T-cell markers and B-cell associated genes.^16^ The second 58-gene panel included type 1-interferon inducible genes and other genes associated with pulmonary tuberculosis.^17^

### Sample collection and RNA-extraction

Within 48 hours of hospital admission, peripheral WB was sampled on PAXgene blood RNA tubes (PreAnalytiX, Switzerland), frozen and stored at −80°C until RNA extraction was performed in 2018 (PAXgene Blood RNA kit, Germany). Total RNA concentration and purity were measured using a Nanodrop spectrophotometer (Thermo Scientific, USA) and ranged between 1.4 and 24.9 μg (mean 7.6 ± 4.2 μg).

### Dual-color-reverse-transcriptase-multiplex-ligation-dependent-probe-amplification (dcRT-MLPA)

For each target sequence, a specific RT primer was designed, located immediately downstream of the left- and right-hand half-probe target sequence. A total RNA of 125 ng was used for reverse transcription, applying MMLV reverse transcriptase (Promega, USA), followed by hybridization of left- and right-hand half-probes to the cDNA at 60°C overnight. Annealed half-probes were ligated and PCR subsequently amplified the ligated product. The remaining steps were performed as described elsewhere.^15,19^ For each of the gene panels, the 156 samples were run on 3 (96-well) plates. The PCR fragments were analyzed on a 3730 capillary sequencer in Gene scan mode (Life Technologies, USA), using GeneMapper version 5.0 (Life Technologies, USA).

### Procalcitonin analysis

PCT was measured in serum sampled within 48 hours of hospital admission using a chemiluminescent assay (ADVIA Centaur BRAHMS PCT, DE), with a functional sensitivity of <0.05 ng/mL.

### Classification of CAP and assignment based on microbiology and PCT

Based on microbiological findings, CAP was classified as; (i) bacterial, (ii) viral, or (iii) mixed viral-bacterial, while patients with unknown microbial etiology were excluded from data analyses. Then, since empirical antibiotic treatment is indicated in all bacterial CAP and we aimed to identify patients where antibiotics could safely be retained, we merged patients with (i) bacterial and (iii) mixed viral-bacterial CAP into bacterial/mixed CAP in relevant analyzes. In a similar approach, based on both microbiological findings and PCT levels, CAP was re-classified as; (i) bacterial-PCT, (ii) viral-PCT, or (iii) mixed viral-bacterial-PCT. In accordance with previously established cut-off levels for serum PCT,^20^ patients with PCT levels of ⩾0.25 ng/mL and detection of a bacterial or mixed viral-bacterial pathogen(s) in microbiological investigations were categorized as bacterial-PCT or mixed viral-bacterial-PCT CAP, while patients with PCT levels <0.25 ng/mL and detection of a viral pathogen in microbiological investigations were categorized as viral-PCT CAP. Patients with PCT levels <0.25 ng/mL and detection of a bacterial/mixed pathogen(s) in microbiological investigations as well as, patients with PCT levels ⩾0.25 ng/mL and detection of a viral pathogen in microbiological investigations were excluded from further analyses.

### Statistical analysis

Differences in clinical characteristics between the study groups were assessed by Pearson’s chi-square test with Yates Continuity Correction or Fisher’s exact test, where appropriate.

For gene expression analysis, GAPDH was used for normalization. For the 11 genes which were present in more than one panel, data from the run with the highest mean expression was used. Further, analysis was carried out for all biomarkers with detectable levels (⩾200 arbitrary units). A 2-step approach was applied for data analysis during identification of host transcriptional signatures: To identify genes differentially expressed between bacterial or bacterial/mixed CAP patients versus viral CAP patients, we entered data for each of the genes included in the dcRT-MLPA panel into univariate logistic regression models to identify potential biomarkers. Then, differentially expressed single gene markers (*P* < .05, no correction for multiple testing applied) were jointly entered into a LASSO regression model. Optimal tuning parameters were found using a cross-validation step, which was repeated 100 times to stabilize results. A predicted probability <0.5 resulted in classification as bacterial or bacterial/mixed CAP and >0.5 resulted in classification as viral CAP. Genes identified in the LASSO regression model were then combined and considered as constituting specific host transcriptional signatures. The diagnostic abilities of the signatures in both objectives were summarized by means of receiver operator characteristics (ROC) curves. Additionally, internal validation by splitting data into training and test sets was performed. Due to the relatively small sample size of study groups and only minor variations in results compared to not performing this step, results of internal validation are presented in Supplemental Figure 1A-D. Analyses were carried out using IBM SPSS version 21 (IBM, Bergen, Norway) and R (R Core Team, 2016),^21^ through the interface RStudio (http://www.rstudio.com/)

## Results

### Baseline characteristics of the study population

**Figure 1.**
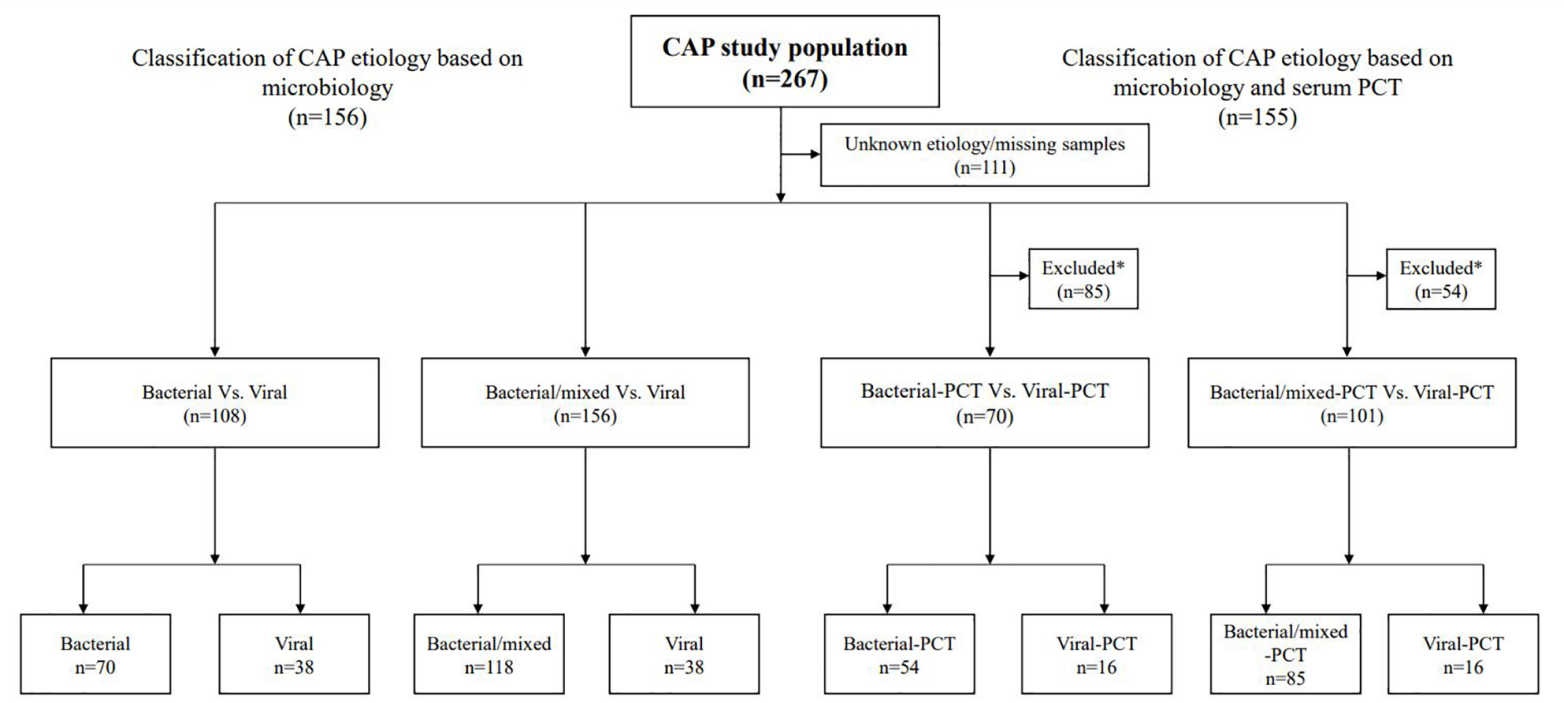
Flow chart for classification of CAP etiology based on microbiological findings and PCT levels. *Procalcitonin (PCT) levels with cut-off levels of 0.25 ng/mL were used to classify bacterial-PCT or bacterial/mixed-PCT CAP (⩾0.25 ng/mL) and viral-PCT CAP (<0.25 ng/mL) in combination with microbiological investigations. Patients who did not meet both the inclusion criteria were excluded from further analysis.

Of the 267 patients included in this prospective cohort study, patients with unknown microbial etiology (n = 100) and missing RNA samples at hospital admission (n = 11) were excluded, leaving an analysis cohort of 156 (Figure 1). The mean time from hospital admission to study inclusion was 0.6 days, and 155 of 156 (99.4%) patients were included within 24 hours. The median age was 64 (25-75th percentile [52-76]) years, 45% were females and 26% were active smokers (Table 1). An extensive microbiological work-up established a bacterial agent in 118 (75.7%) of whom 70 (44.9%) were classified as bacterial CAP and 48 (30.8%) were classified as mixed viral-bacterial CAP (Supplemental Table 1). A viral cause was established in 38 (24.4%) patients. Preexisting comorbidities were frequent as 99 (63.5%) patients had ⩾1 comorbid condition, cardiovascular disease (heart failure, coronary heart disease, cerebrovascular disease, and/or peripheral artery disease) and chronic obstructive pulmonary disease being the most common (Table 1).

**Table 1.** Baseline characteristics of 156 hospitalized patients with community-acquired pneumonia.

Characteristics	Patients	Missing data
Demographics		
Age (years)	64 (52-76)	
Male gender, n (%)	85 (54.5)	
Active smoker, n (%)	40 (25.8)	1
Comorbid conditions, n (%)		
Cardiovascular disease^a^	42 (26.9)	
COPD	35 (22.4)	
Immunocompromised ^b^	27 (17.3)	
Autoimmune disease^c^	30 (12.2)	
Diabetes mellitus	22 (14.1)	
Renal disease	21 (13.5)	
Etiology, n (%)		
Bacterial	70 (44.9)	
Viral	38 (24.4)	
Viral-bacterial	48 (30.7)	
Vaccination status, n (%)		
Influenza vaccination (<1 y)	38 (33.0)	41
Pneumococcal vaccination (<10 y)	14 (12.1)	40
Disease severity, n (%)		
CURB-65 ⩾3	60 (39.0)	2
ICU admission	26 (16.7)	

Abbreviations: COPD, chronic obstructive pulmonary disease; CURB-65, confusion, urea, respiratory rate, blood pressure, age ⩾ 65; ICU, intensive care unit.

Data are presented as medians (25th-75th percentile) or No. (%).

a Heart failure, coronary heart disease, cerebrovascular disease and/or peripheral artery disease.

b Rheumatoid arthritis, systemic lupus erythematosus, inflammatory bowel disease, autoimmune hepatitis, Sjogren’s disease, psoriasis.

c Primary or acquired immunodeficiency, active malignancy, immunosuppressive drugs.

### Identification of transcriptional signatures based on microbiological testing

#### A 9-transcript gene signature discriminated bacterial CAP from viral CAP

Potential gene biomarkers were first identified in bacterial and viral CAP based on microbiological investigations, as these groups can be assumed to have more distinct immune profiles allowing for more accurate classification and thus the “purest” host transcriptional signatures. Univariate logistic regression analysis identified 34 genes differentially expressed between patients with bacterial CAP and viral CAP. These 34 genes were entered into a LASSO regression model, resulting in a 9-transcript gene signature comprising *CCL3*, *CD3E*, *CXCL13*, *GUSB*, *GZMA*, *IFI44*, *IL5*, *IL13*, and *TNF*. The 9-transcript signature correctly classified 66 of 70 bacterial CAP cases and 24 of 38 viral CAP cases corresponding to an area under the curve (AUC) of 0.91 (95% CI 0.85-0.96) with a sensitivity of 94.3% (95% CI 86.0-98.4) and a specificity of 63.2% (95% CI 46.0-78.2, Figure 2A).

**Figure 2A.**
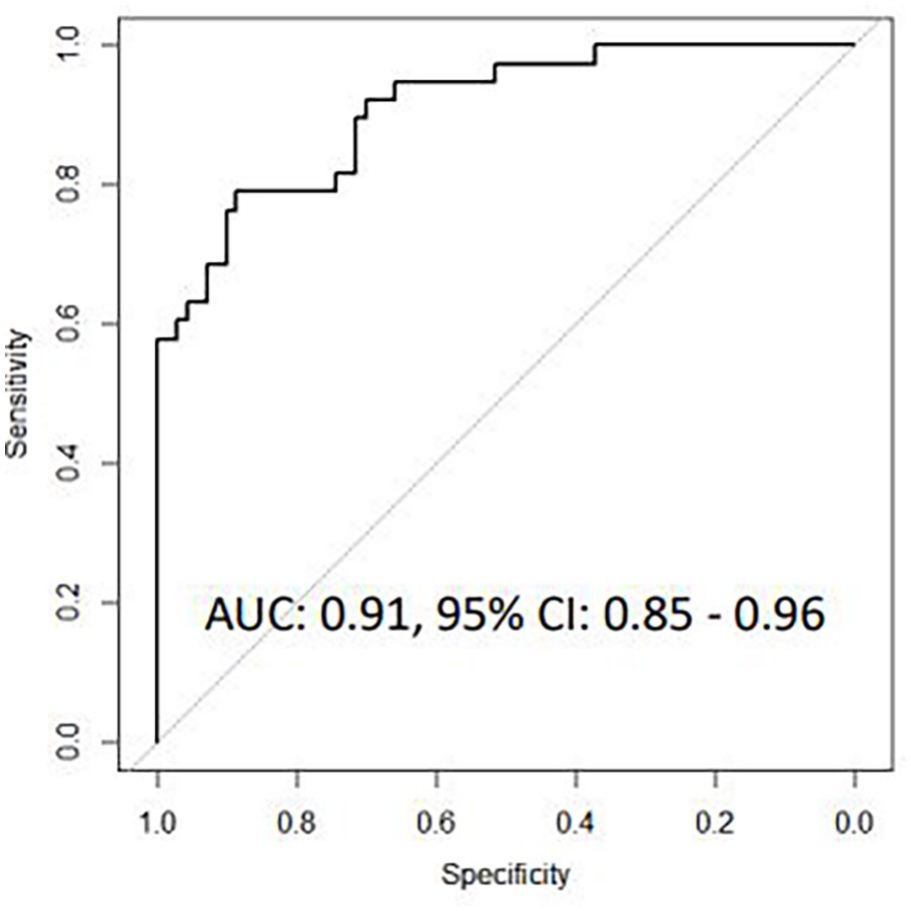
Receiver operating characteristic curves for host gene signatures for discriminating bacterial CAP from viral CAP based on microbiological findings.

#### A 10-transcript gene signature discriminated bacterial/mixed CAP from viral CAP

Univariate logistic regression analysis identified 32 genes that were differentially expressed between patients with bacterial/mixed CAP and viral CAP. These 32 genes where then entered into a LASSO regression model, resulting in a 10-transcript signature comprising *CCL3*, *CCL5*, *CD3E*, *CXCL13*, *FLCN1*, *GUSB*, *GZMA*, *IFI44*, *IL13*, and *TBX21*. This 10-transcript signature correctly classified 113 of 118 bacterial CAP cases and 17 of 38 viral CAP cases, corresponding to an AUC of 0.91, 95% CI 0.81 to 0.96), a sensitivity of 95.8% (95% CI 90.4-98.6) and a specificity of 44.7% (95% CI 28.6-61.7, Figure 2B). In the 9-transcript and 10-transcript gene signatures identified based on microbiological investigations, 7 genes (*CCL3*, *CD3E*, *CXCL13*, *GUSB*, *GZMA*, *IFI44*, and *IL13)* overlapped.

**Figure 2B.**
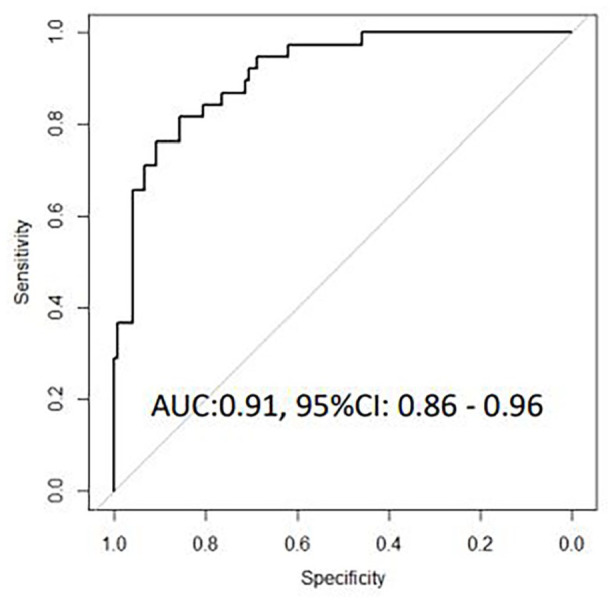
Receiver operating characteristic curves for host gene signatures for discriminating bacterial/mixed CAP from viral CAP based on microbiological findings.

### Identification of transcriptional signatures based on microbiological testing and PCT

#### A 13-transcript gene signature discriminated bacterial-PCT CAP from viral-PCT CAP

We used a similar approach for identification of host transcriptional signatures in CAP etiology based on both microbiological investigations and PCT levels. Univariate logistic regression analysis identified 46 genes differentially expressed between patients with bacterial-PCT CAP and viral-PCT CAP. These 46 genes where then entered into a LASSO regression model, resulting in a 13-transcript gene signature comprising *ABR, BMP6, CCL4, CXCL10, GNLY, GUSB, IFI35, IFI44L, IL13, NLRC4, NLRP3, NOD2*, and *TNF*. The 13-transcript signature correctly classified all 54 of 54 bacterial-PCT CAP cases and 13 of 16 viral-PCT CAP cases corresponding to an AUC of 1.00 (1.00-1.00) with a sensitivity of 100.0% (95% CI 93.4-100.0) and a specificity of 81.3% (95% CI 54.4-96.0, Figure 2C).

**Figure 2C.**
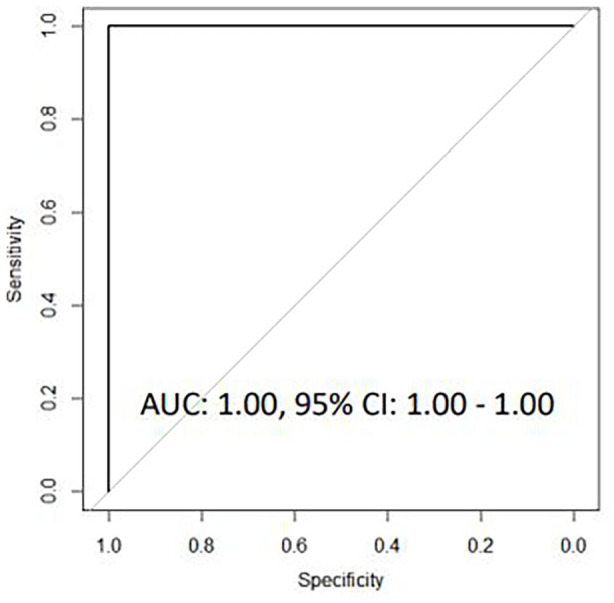
Receiver operating characteristic curves for host gene signatures for discriminating bacterial-PCT CAP from viral-PCT CAP based on microbiological findings and serum PCT levels.

#### A 7-transcript gene signature discriminated bacterial/mixed-PCT CAP from viral-PCT CAP

Univariate logistic regression analysis identified 28 genes differentially expressed between patients with bacterial/mixed-PCT CAP and viral-PCT CAP. These 28 genes where then entered into a LASSO regression model, resulting in a 7-transcript signature comprising *BLR1*, *CCL3*, *CCL4*, *CD4*, *GNLY*, *GUSB*, and *IL13*. This 7-transcript signature correctly classified 84 of 85 bacterial-PCT CAP cases and 6 of 16 viral-PCT CAP cases, corresponding to an AUC of 0.93, (95% CI 0.87-0.98), a sensitivity of 98.8% (95% CI 93.6-99.8) and a specificity of 37.5% (95% CI 15.2-64.6, Figure 2D). In the 13-transcript and 7-transcript gene signatures identified based on both microbiological investigations and PCT levels, 4 genes (*CCL4*, *GUSB*, *IFI44*, and *IL13*) overlapped.

**Figure 2D.**
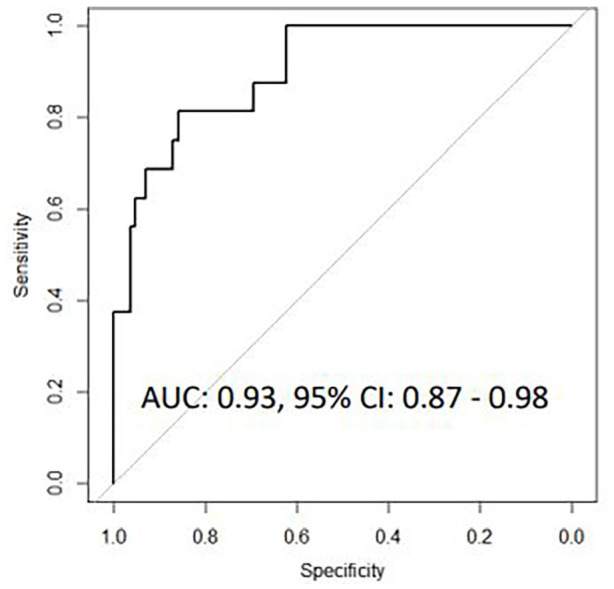
Receiver operating characteristic curves for host gene signatures for discriminating bacterial/mixed-PCT CAP from viral-PCT CAP based on microbiological findings and serum PCT levels.

## Discussion

In this study, we applied the robust and low-cost method dcRT-MLPA to identify host transcriptional gene signatures as potential markers for determining etiology of CAP. Firstly, a 9-transcript signature with an AUC of 0.91 for discriminating between bacterial and viral etiology in CAP was identified. Secondly, when mixed viral-bacterial CAP was compared with viral CAP, a 10-transcript signature provided a very high discriminatory accuracy of 91% (AUC 0.91). In both populations, sensitivity was high (94.3% and 95.8% respectively), with most bacterial CAP cases correctly classified, but with lower specificity. Notably, the identified signatures were highly overlapping, comprising seven common genes (*CCL3*, *CD3E*, *CXCL13*, *GUSB*, *GZMA*, *IFI44*, *IL13*). Given the absence of a reliable gold standard for CAP etiology classification, we further hypothesized that the combined use of extensive microbiological testing and established PCT cut-off levels could provide a more robust classification of CAP etiology, associated with particularly distinct immune profiles. A 13-transcript gene signature identified in the subpopulation comparing bacterial-PCT versus viral-PCT CAP correctly classified all 54 bacterial-PCT CAP cases and 13 of 16 viral-PCT CAP, with an AUC of 1.00, a sensitivity of 100% and specificity of 81.3%. In the more heterogeneous subpopulation of bacterial/mixed CAP-PCT compared with viral-PCT CAP, a 7-transcript signature discriminated bacterial/mixed infection from viral infection also with excellent accuracy (AUC 0.93). In summary, the accuracy of the transcriptional signatures for discriminating bacterial from viral CAP far exceeded established protein biomarkers in current clinical use.^9^ Although warranting validation in other studies, our findings are promising and potentially relevant for future non-sputum based POC diagnostic tools for adult CAP.

In a microarray analysis of 118 patients with LRTIs, Suarez et al^12^ found an excellent accuracy (AUC 0.91 and 0.96) of a 10-transcript signature for discriminating bacterial from viral LRTI. Similarly, in another microarray-based study from Tsalik et al^13^, host gene signatures with excellent accuracy (AUC 0.90-0.98) for discriminating bacterial from viral RTIs were described, with a non-infectious illness control group also included. Later large-scale LRTI studies have corroborated these findings with equally impressive diagnostic accuracies.^14,22^ Beyond adult populations with LRTIs, there is a growing body of evidence for host gene signatures providing diagnostic information in numerous infectious conditions. Most relevant, diagnostic transcriptional signatures have been identified in sepsis,^23-25^ pulmonary tuberculosis,^26-29^ and pediatric populations with febrile illness, influenza A, and respiratory syncytial virus.^30-32^ Thus, the results from our study are in line with previous observations and support the potential clinical benefit of transcriptional host signatures in LRTIs.

Current diagnostic approaches in CAP focus on pathogen detection and characterization using traditional methods (ie, bacterial cultures, urinary antigen assays, serology) and PCR, supported by protein biomarkers for clinical decision-making.^1^ Although molecular tests represent major advancements for increasing the diagnostic yield in CAP, these methods have important limitations and do not currently allow for antibiotic stewardship.^33^ Moreover, PCR-based methods and especially multiplex diagnostic platforms are restricted in terms of breadth of pathogens detected, as one needs an a-priori knowledge of pathogens to be tested. Hence, the presence of one pathogen does not exclude the presence of other undetected pathogens. The identified pathogens may represent varying clinical significance; from detection of asymptomatic carriage or shedding of respiratory viruses to the causative pathogen, although this at least in part can be avoided by use of semiquantitative methods for grouping/binning of microbiological findings.^33,34^ Appropriate sampling from the respiratory tract is challenging, as samples can be contaminated by commensals from the nasal and oropharyngeal microbiotas.^7^ Additionally, invasive sampling from the respiratory tract may be harmful to patients (eg, by inducing pneumonia),^35^ while also carrying a risk of pathogen exposure for health-care workers, as underlined by the Covid-19 pandemic.^36^

As an alternative, or ideally, as a complementary tool, host transcriptional signatures may be identified in peripheral whole blood samples, thus offering a simpler strategy for determining etiology in CAP, as well as other infectious conditions. Most studies to date have used large-scale analyses such as RNA sequencing or microarray for signature identification in LRTIs,^11-14^ thus not representing realistic alternatives for rapid point-of-care testing in the clinical setting. In this study, we used the dcRT-MLPA, an inexpensive method with the potential of analyzing up to 100 genes per sample and a comparable dynamic range and sensitivity to real-time qPCR and RNA sequencing.^15^ We demonstrate that a high accuracy for discriminating bacterial from viral CAP is achievable in a medium-scale analysis, thereby lowering costs required for host gene signature identification in CAP. By use of established, albeit controversial cut-off levels for PCT for CAP classification, both sensitivity and specificity of the signatures increased, resulting in excellent diagnostic accuracy. Although the ability of PCT to discriminate bacterial from viral CAP is not absolute,^4^ our study suggests that a combination of PCT and transcriptional signatures may have a potential role in the identification of viral from mixed and bacterial CAP and thus, may represent a promising antibiotic stewardship tool. A low PCT value combined with a “viral” host gene signature may provide substantial support for the clinician when faced with a dilemma of prescribing or withholding antibacterial therapy. Still, the turnaround time for the dcRT-MLPA is 72 hours; thus, it is not suited for clinical use at present. In order to identify CAP patients where antibiotic treatment could safely be retained, identified signatures need to be translated into a point-of-care test with a shorter turnaround time, without loss of diagnostic accuracy. Encouragingly, Lydon et al recently published results from a RT-PCR test in acute RTI patients based on gene signatures previously identified in a microarray study, with accuracy of 88%, 84%, and 82% for discriminating bacterial, viral and noninfectious illness, respectively.^37^ In addition, a point-of-care test based on a 29-gene biomarker signature is under development for early diagnosis of sepsis with the aim of extrapolating this method to other infectious conditions.^38^ In light of recent advances, the Infectious Diseases Society of America’s Diagnostics Committee has recommended the combination of host signatures, simultaneous pathogen detection and antibiotic stewardship as a focus area for future diagnostic respiratory studies.^33^

This study has some limitations. Firstly, the identified host gene signatures were obtained in a single-center hospital cohort; thus, our results lack external validation. However, internal validation based on dividing into training and test sets was indeed applied in the LASSO step by means of cross validation with only minor variations in results. Nevertheless, it is necessary to validate our findings in other CAP cohorts. Further, we did not include a control group of patients with noninfectious causes of hospital admission. We acknowledge that such a control group would strengthen our findings. Lastly, the transcriptional signatures determined in our study were derived from peripheral WB samples and our results do not necessarily reflect the local infection response. However, these peripheral WB signatures probably reflect general inflammatory responses induced by bacterial, mixed viral-bacterial infection or viral infection and may therefore be equally or more relevant for clinical decision-making in the acute-phase of CAP. Peripheral blood samples are frequently obtained in the clinical routine and have a low cost and complication rate. Thus, we rather consider this a strength of our study.

## Conclusion

In conclusion, we have identified host transcriptional signatures with high accuracy for discriminating bacterial CAP or mixed viral-bacterial infections from viral CAP. Further refinement and validation of the signatures are warranted in other CAP cohorts, to enable their use for guiding antibiotic treatment in hospitalized patients with CAP. Our findings give promise of a rapid and reliable adjunctive tool for differentiating bacterial and viral etiology and a potential new tool for antibiotic stewardship.

## Supplemental Material

sj-docx-1-bmi-10.1177_11772719221099130 – Supplemental material for Host Transcriptional Signatures Predict Etiology in Community-Acquired Pneumonia: Potential Antibiotic Stewardship Tools Click here for additional data file. Supplemental material, sj-docx-1-bmi-10.1177_11772719221099130 for Host Transcriptional Signatures Predict Etiology in Community-Acquired Pneumonia: Potential Antibiotic Stewardship Tools by William W Siljan, Dhanasekaran Sivakumaran, Christian Ritz, Synne Jenum, Tom HM Ottenhoff, Elling Ulvestad, Jan C Holter, Lars Heggelund and Harleen MS Grewal in Biomarker Insights
