# Supplementary material for: Host Transcriptional Signatures Predict Etiology in Community-Acquired Pneumonia: Potential Antibiotic Stewardship Tools
Source: Biomark Insights. 2022 Jun 6;17:11772719221099130. doi: 10.1177/11772719221099130 (PMC9174553; doi:10.1177/11772719221099130)
Supplement: sj-docx-1-bmi-10.1177_11772719221099130 – Supplemental material for Host Transcriptional Signatures Predict Etiology in Community-Acquired Pneumonia: Potential Antibiotic Stewardship Tools [file sj-docx-1-bmi-10.1177_11772719221099130.docx]

**Supplementary Text 1. The inclusion process for the study population.**

Adult patients (aged ≥18 years) with suspected pneumonia admitted to Medical Department, Drammen Hospital, Vestre Viken Hospital Trust in Norway were consecutively recruited between January 2008 and January 2011. A total of 320 patients were screened within the first 48 hours of admission. Of these, 33 (10%) patients were excluded (based on the predefined criteria) for the following reasons: previous hospitalization within past ≤2 weeks (2 patients), chest radiograph was not performed (1), no new infiltrate was detected (19), non- infectious cause of pulmonary infiltrate and/or bronchial obstruction was revealed (7), and fever was not documented (4). A total of 287 patients (90% of the screened population) were eligible. Of these, 4 (1%) patients did not consent to enter the study. Sixteen patients who entered the study were subsequently withdrawn (6%, 16 of 287 patients) for the following reasons: consent withdrawal (1 patient), previous participation (2), reduced cooperation (2), missing or incorrect ID on case record form (3), inadequate sampling (2), and initial positive chest radiographic findings failed by review of radiologist (6). Of the remaining 267 patients who were included in the study and followed up, 8 (3%) died in the hospital.

**Supplementary Figure 1A-D**

Based on microbiological findings, CAP was classified as; (i) bacterial, (ii) viral, or (iii) mixed viral-bacterial, while patients with unknown microbial etiology were excluded from data analyses. Then, since empirical antibiotic treatment is indicated in all bacterial CAP and we aimed to identify patients where antibiotics could safely be retained, we merged patients with (i) bacterial and (iii) mixed viral-bacterial CAP into bacterial/mixed CAP in relevant analyzes. For internal validation patients were randomly assigned to training (2/3 of patients) and test sets (1/3 of patients) for the comparison of gene expression between study groups based on microbiological etiology. In a similar approach, based on both microbiological findings and PCT levels, CAP was re-classified as; (i) bacterial-PCT, (ii) viral-PCT, or (iii) mixed viral-bacterial-PCT and patients were randomly assigned to training (2/3 of patients) and test sets (1/3 of patients). In accordance with previously established cut-off levels for serum PCT, patients with PCT levels of ≥0.25 ng/mL and detection of a bacterial or mixed viral-bacterial pathogen(s) in microbiological investigations were categorized as bacterial-PCT or mixed viral-bacterial-PCT CAP, while patients with PCT levels <0.25 ng/mL and detection of a viral pathogen in microbiological investigations were categorized as viral-PCT CAP. Patients with PCT levels <0.25 ng/mL and detection of a bacterial/mixed pathogen(s) in microbiological investigations as well as patients with PCT levels ≥0.25 ng/mL and detection of a viral pathogen in microbiological investigations were excluded from further analyses.


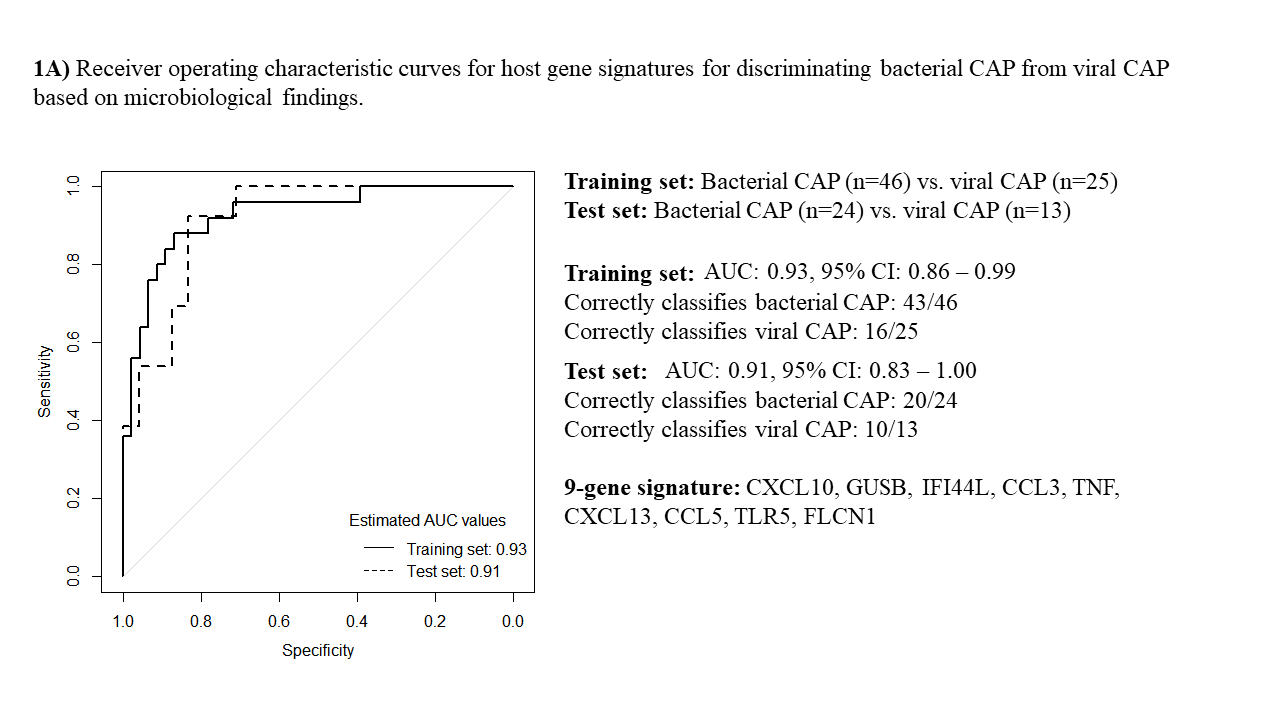


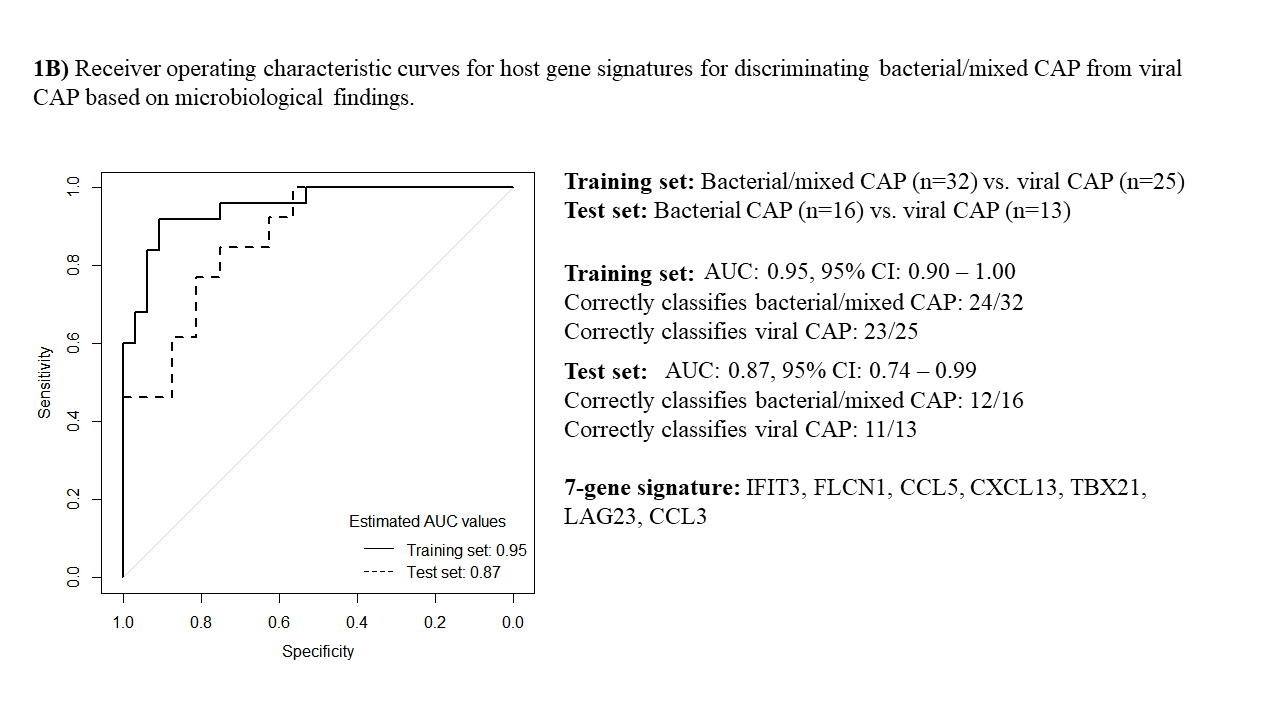


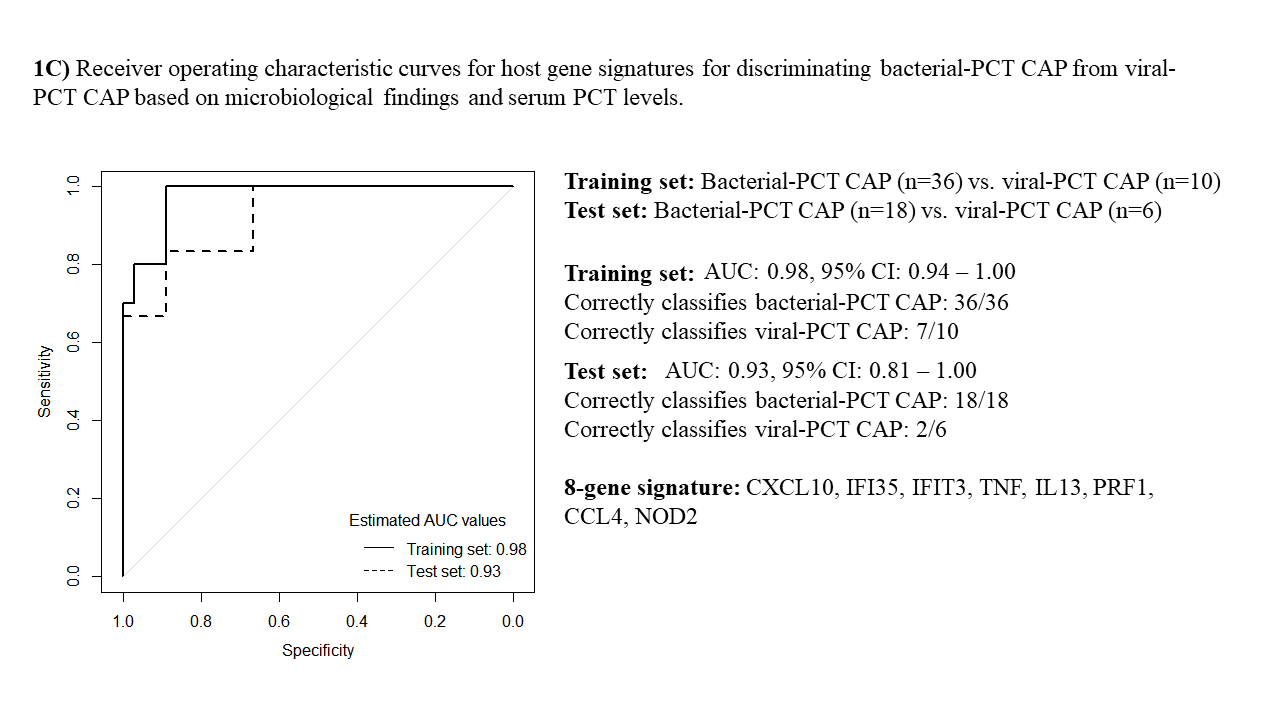


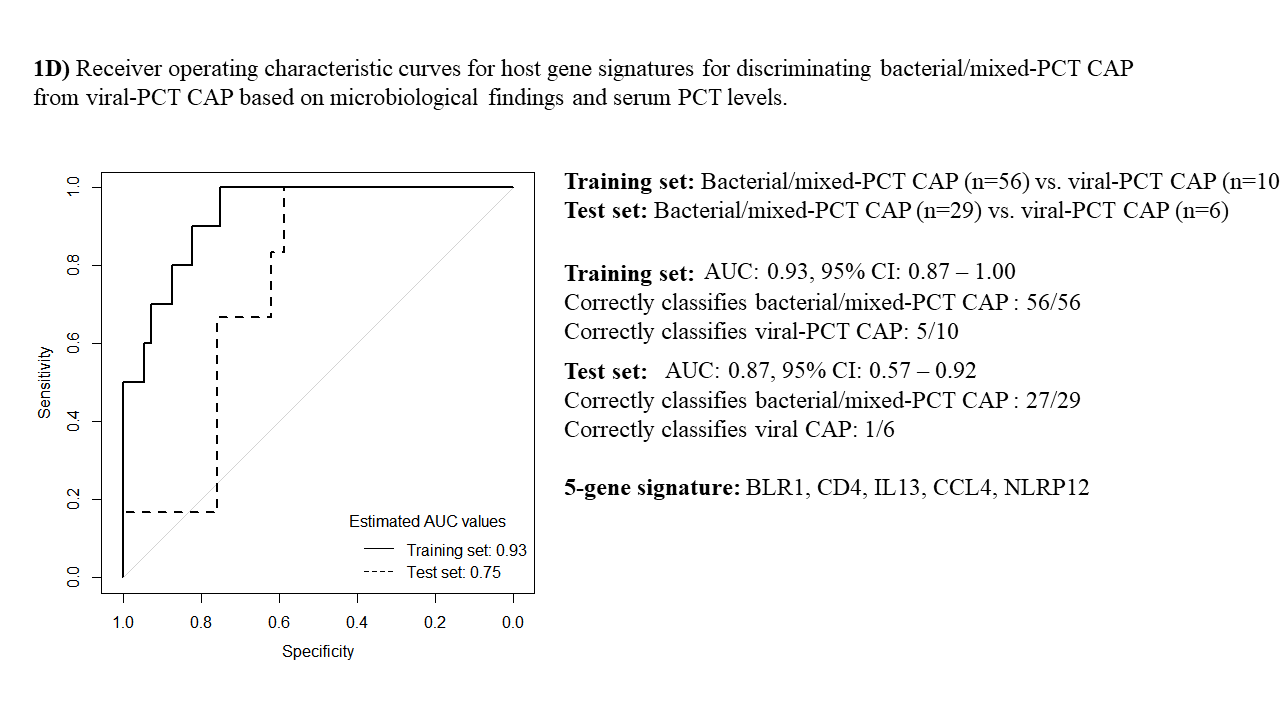


**Supplementary Table 1 Microbial findings in 267 hospitalized patients with community-acquired pneumonia**

| Bacterial pathogens | No. (%) with positive findings |  | Viral pathogens | No. (%) with positive findings |
| --- | --- | --- | --- | --- |
| *Streptococcus pneumoniae* | 81 (30) |  | Influenza viruses^d^ | 40 (15) |
| *Bordetella pertussis* | 15 (6) |  | Rhinovirus | 32 (12) |
| *Haemophilus influenzae* | 14 (5) |  | Parainfluenza viruses^e^ | 8 (3) |
| *Mycoplasma pneumoniae* | 10 (4) |  | Respiratory syncytial virus | 7 (3) |
| *Chlamydophila pneumoniae* | 7 (3) |  | Metapneumovirus | 7 (3) |
| *Legionella pneumophila* | 7 (3) |  | Enterovirus | 5 (2) |
| *Enterobacteriacea^a^* | 6 (2) |  | Adenovirus | 1 (0.4) |
| *Moraxella catarrhalis* | 5 (2) |  |  |  |
| *Miscellaneous^b^* | 3 (1) |  |  |  |
| *Haemophilus parainfluenzae* | 2 (1) |  |  |  |
| Total^c^ | 126 (47) |  | Total^c^ | 92 (34) |

^a^ Include either of the following: E. coli, P. aeruginosa or Enterobacter species.

^b^ Include either of the following: Group A streptococcus, Prevotella spp., Dialister pneumosintes, and Group A streptococcus.

^c^ No. of patients does not add up to no. of pathogens because some patients had multiple pathogens detected

^d^ Influenza A virus and Influenza B virus.

^e^ Parainfluenza virus type 1, type 2, and type 3.

| **Supplementary Table S2 The distribution of the 156 genes in the two gene panels used in the dc-RT MLPA** | |
| --- | --- |
| **Gene** | **Panel 1** |
|  | **Gene Name** |
| **AIRE** | Autoimmune regulator |
| **AREG** | Amphiregulin |
| **BPI** | Bactericidal/Permeability-Increasing Protein |
| **CAMTA1** | Calmodulin Binding Transcription Activator 1 |
| **CCL2** | C-C Motif Chemokine Ligand 2 |
| **CCL22** | C-C Motif Chemokine Ligand 22 |
| **CCL4** | C-C Motif Chemokine Ligand 4 |
| **CCL5** | C-C Motif Chemokine Ligand 5 |
| **CCR7** | C-C Motif Chemokine Receptor 7 |
| **CD209** | CD209 Molecule |
| **CD3E** | CD3e Molecule |
| **CD8A** | CD8a Molecule |
| **CLEC7A** | C-Type Lectin Domain Family 7 Member A |
| **CXCL13** | C-X-C Motif Chemokine Ligand 13 |
| **FCGR1A** | Fc Fragment of IgG Receptor Ia |
| **FLCN1** | Folliculin |
| **GATA3** | GATA Binding Protein 3 |
| **GNLY** | Granulysin |
| **GZMA** | Granzyme A |
| **GZMB** | Granzyme B |
| **IFNG** | Interferon Gamma |
| **IL2** | Interleukin2 |
| **IL5** | Interleukin 5 |
| **IL9** | Interleukin 9 |
| **IL10** | Interleukin 10 |
| **IL13** | Interleukin 13 |
| **IL15** | Interleukin 15 |
| **IL12A** | Interleukin 12A |
| **IL17A** | Interleukin 17A |
| **IL23A** | Interleukin 23A |
| **IL1B** | Interleukin 1B |
| **IL12B** | Interleukin 12B |
| **LAG3** | Lymphocyte Activating 3 |
| **MRC1** | Mannose Receptor C-Type 1 |
| **MRC2** | Mannose Receptor C-Type 2 |
| **NEDD4L** | Neural Precursor Cell Expressed, Developmentally Down-Regulated 4-Like, E3 Ubiquitin Protein Ligase |
| **NLRC4** | NLR Family CARD Domain Containing 4 |
| **NLRP1** | NLR Family Pyrin Domain Containing 1 |
| **NLRP2** | NLR Family Pyrin Domain Containing 2 |
| **NLRP3** | NLR Family Pyrin Domain Containing 3 |
| **NLRP4** | NLR Family Pyrin Domain Containing 4 |
| **NLRP6** | NLR Family Pyrin Domain Containing 6 |
| **NLRP7** | NLR Family Pyrin Domain Containing 7 |
| **NLRP10** | NLR Family Pyrin Domain Containing 10 |
| **NLRP11** | NLR Family Pyrin Domain Containing 11 |
| **NLRP12** | NLR Family Pyrin Domain Containing 12 |
| **NLRP13** | NLR Family Pyrin Domain Containing 13 |
| **NOD1** | Nucleotide Binding Oligomerization Domain Containing 1 |
| **NOD2** | Nucleotide Binding Oligomerization Domain Containing 2 |
| **PRF1** | Perforin 1 |
| **PTPRCv1** | Protein tyrosine phosphatase receptor type Cv1 |
| **PTPRCv2** | protein tyrosine phosphatase receptor type Cv2 |
| **RORC** | RAR Related Orphan Receptor C |
| **TAGAP** | T-Cell Activation RhoGTPase Activating Protein |
| **TBC1D7** | TBC1 Domain Family Member 7 |
| **TBX21** | T-Box 21 |
| **TGFB1** | Transforming Growth Factor Beta 1 |
| **TLR1** | Toll Like Receptor 1 |
| **TLR2** | Toll Like Receptor 2 |
| **TLR3** | Toll Like Receptor 3 |
| **TLR4** | Toll Like Receptor 4 |
| **TLR5** | Toll Like Receptor 5 |
| **TLR6** | Toll Like Receptor 6 |
| **TLR7** | Toll Like Receptor 7 |
| **TLR8** | Toll Like Receptor 8 |
| **TLR9** | Toll Like Receptor 9 |
| **TLR10** | Toll Like Receptor 10 |
| **TNF** | Tumor Necrosis Factor |
| **TNFRSF18** | TNF Receptor Superfamily Member 18 |
| **TWIST1** | Twist Family BHLH Transcription Factor 1 |
| **ZNF331** | Zinc Finger Protein 331 |
| **ZNF532** | Zinc Finger Protein 532 |
|  | **Panel 2** |
| **ASAP1** | ArfGAP With SH3 Domain, Ankyrin Repeat And PH Domain 1 |
| **B2M** | Beta-2-Microglobulin |
| **BMP6** | Bone Morphogenetic Protein 6 |
| **CCL11** | C-X-C Motif Chemokine Ligand 11 |
| **CCL3** | C-C Motif Chemokine Ligand 3 |
| **CD274** | CD274 Molecule |
| **CX3CL1** | C-X3-C Motif Chemokine Ligand 1 |
| **CXCL9** | C-X-C Motif Chemokine Ligand 9 |
| **CXCL10** | C-X-C Motif Chemokine Ligand 10 |
| **DSE** | Dermatan Sulfate Epimerase |
| **EGF** | Epidermal Growth Factor |
| **GBP1** | Guanylate Binding Protein 1 |
| **GBP2** | Guanylate Binding Protein 2 |
| **GBP5** | Guanylate Binding Protein 5 |
| **GUSB** | Glucuronidase Beta |
| **HCK** | HCK Proto-Oncogene, Src Family Tyrosine Kinase |
| **HPRT** | Hypoxanthine Phosphoribosyltransferase 1 |
| **IFI6** | Interferon Alpha Inducible Protein 6 |
| **IFI16** | Interferon Gamma Inducible Protein 16 |
| **IFI35** | Interferon Induced Protein 35 |
| **IFI44** | Interferon Induced Protein 44 |
| **IFI44L** | Interferon Induced Protein 44 Like |
| **IFIH1** | Interferon Induced With Helicase C Domain 1 |
| **IFIT2** | Interferon Induced Protein With Tetratricopeptide Repeats 2 |
| **IFIT3** | Interferon Induced Protein With Tetratricopeptide Repeats 3 |
| **IFIT5** | Interferon Induced Protein With Tetratricopeptide Repeats 5 |
| **IFITM3** | Interferon Induced Transmembrane Protein 3 |
| **IL6** | Interleukin 6 |
| **INDO** | Indoleamine 2,3-Dioxygenase 1 |
| **IRF7** | Interferon Regulatory Factor 7 |
| **KIF1B** | Kinesin Family Member 1B |
| **LYN** | LYN Proto-Oncogene, Src Family Tyrosine Kinase |
| **OAS1** | 2'-5'-Oligoadenylate Synthetase 1 |
| **OAS2** | 2'-5'-Oligoadenylate Synthetase 2 |
| **OAS3** | 2'-5'-Oligoadenylate Synthetase 3 |
| **SLAMF7** | SLAM Family Member 7 |
| **SOCS1** | Suppressor Of Cytokine Signaling 1 |
| **STAT1** | Signal Transducer And Activator Of Transcription 1 |
| **STAT2** | Signal Transducer And Activator Of Transcription 2 |
| **TAP1** | Transporter 1, ATP Binding Cassette Subfamily B Member |
| **TAP2** | Transporter 2, ATP Binding Cassette Subfamily B Member |
| **TNIP1** | TNFAIP3 Interacting Protein 1 |
| **VEGF** | Vascular Endothelial Growth Factor A |
|  |  |
| The repeated genes (genes present in more than one panel) are presented in the panel where the gene gave the highest mean expression in all study subjects. | |
